# The effect of an intercalated BSc on subsequent academic performance

**DOI:** 10.1186/1472-6920-11-76

**Published:** 2011-10-03

**Authors:** Nishanthan Mahesan, Siobhan Crichton, Hannah Sewell, Simon Howell

**Affiliations:** 1 School of Medicine, Guy's Campus, King's College London, London, UK; 2 Department of Public Health Sciences, Capital House, King's College London, London, UK; 3 Henriette Raphael House, Guy's Campus, King's College London, London, UK; 4 Hodgkin Building, Guy's Campus, King's College London, London, UK

## Abstract

**Background:**

The choice of whether to undertake an intercalated Bachelor of Science (BSc) degree is one of the most important decisions that students must make during their time at medical school. An effect on exam performance would improve a student's academic ranking, giving them a competitive edge when applying for foundation posts.

**Methods:**

Retrospective data analysis of anonymised student records. The effects of intercalating on final year exam results, Foundation Programme score, application form score (from white-space questions), quartile rank score, and success with securing Foundation School of choice were assessed using linear and ordered logistic regression models, adjusted for course type, year of graduation, graduate status and baseline (Year 1) performance.

**Results:**

The study included 1158 students, with 54% choosing to do an intercalated BSc, and 9.8% opting to do so at an external institution. Doing an intercalated BSc was significantly associated with improved outcome in Year 5 exams (P = 0.004). This was irrespective of the year students chose to intercalate, with no significant difference between those that intercalated after years 2, 3 and 4 (p = 0.3096). There were also higher foundation application scores (P < 0.0001), academic quartile scores (P = 0.0003) and resultant overall foundation scores (P < 0.0001) in intercalated students. These students also had improved success with securing their first choice Foundation School (p = 0.0220). Participants who remained at the institution to intercalate in general performed better than those that opted to intercalate elsewhere.

**Conclusions:**

Doing an intercalated BSc leads to an improvement in subsequent exam results and develops the skills necessary to produce a strong foundation programme application. It also leads to greater success with securing preferred Foundation School posts in students. Differences between internally- and externally-intercalating students may be due to varying course structures or greater challenge in adjusting to a new study environment.

## Background

The choice of whether to undertake an intercalated Bachelor of Science (BSc) degree is one of the most important decisions that students must make during their time at medical school. In addition to broadening research skills, it also provides the opportunity to publish work and distinguish oneself in a competitive field [1]. It is reported that the most common reason students choose to do an intercalated BSc is to improve career prospects [2]. However, these benefits must be weighed against potential drawbacks; the financial burden of spending an additional year at university and possible difficulties of returning to medical studies, particularly if one has intercalated within the clinical years. With the introduction of the NHS Bursary, intercalating medical students in the UK are eligible for financial assistance one year earlier than those that do not intercalate, which may ease their hardship and reduce the debt that they may accrue over this additional year. However, deferring salaried work for another year results in the loss of potential earnings, which may deter many students, particularly those from the poorest backgrounds. There is therefore a need for individuals to weigh up these various factors. Despite extensive discussion, it is reported that many students remain unclear about the true benefits of intercalating, with recent calls for these to be better defined and presented in a way that allows students to make a more informed decision about whether it would suit them and their career aspirations [3].

**Figure 1:**
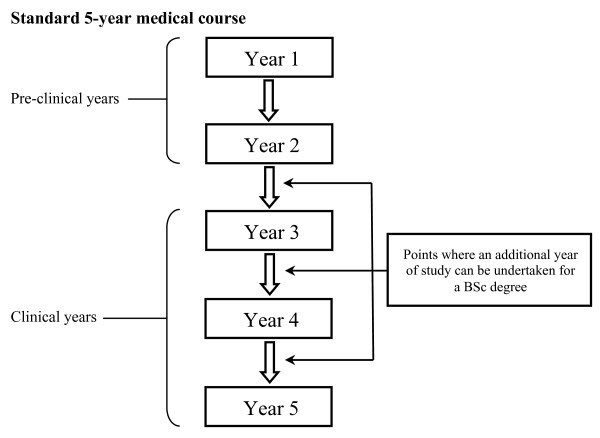
**Standard 5-year medical course**. Each box represents a separate year of study. Year 5 represents the final year of medical studies. Applications to Foundation Schools are made during Year 5, and the examination results from the preceding years are used to determine academic rankings.

**Figure 2:**
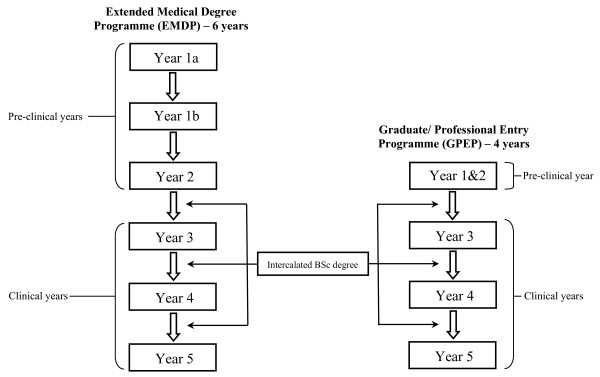
**Other available medical programmes**. Both the EMDP and GPEP courses cover the same material as the standard 5-year course and lead to the same MBBS qualification. Year 1 of the standard medical course is covered over 2 years on the extended programme, leading to a 6-year course (excluding the optional intercalated BSc year). Years 1 and 2 of the standard medical course are covered in one year in the graduate entry course, leading to a 4-year course.

Figures 1 and 2 outline the structures of the medical courses available. Following medical school, graduates in the UK enter the Foundation Programme, a two-year training programme that builds on the skills and competencies developed during undergraduate studies and prepares them for speciality training. Applications are made to Foundation Schools, and allocations determined by an individual's overall score, based on quartile and application score. The former is related to academic performance in relation to cohort, while the latter is the total derived from answering a set of white-space questions, which centre on providing examples of core competencies such as professionalism, coping with pressure, prioritisation skills, communication and teamwork. Allocation to the most popular Foundation Schools is fiercely competitive, and with increasing applications from non-UK graduates, there is more emphasis than ever before on maximising one's overall Foundation Programme score.

Studies have investigated the academic benefits of doing an additional degree, with suggestions that it may improve exam results in subsequent years. While an improved performance on exams may be perceived as a short-term benefit, this may actually have far greater significance. Exam results form a crucial part of the academic ranking of students for the national Foundation Programme, and an improved exam performance would lead to a higher ranking and place students in a better position to secure their Foundation School of preference. A significant proportion of students found undertaking an intercalated degree to be useful for the rest of their undergraduate course [4]. However, such a subjective measure may not necessarily correlate with improved exam performance. Another study found that students who intercalated in pathology at Edinburgh performed better in the remainder of the course compared to those that did not intercalate [5]. Since this study was conducted at a time when pathology had a more prominent role in medical examinations it may not be indicative of the effect of intercalating on subsequent academic performance with current medical curricula. In a study examining the short-term benefits of intercalating, no consistent advantage on subsequent performance in clinical exams could be found [6]. With a sample size of only 14 students, the conclusions from this study may not be representative. A more recent study found that doing an intercalated BSc was associated with improved marks in subsequent assessments at medical school, at an institution where 18% of the sample investigated, chose to intercalate [7]. Using third year performance as a baseline, retrospective analysis of medical exam results at the University of Aberdeen, showed intercalated students performed better in subsequent years [7]. This is a noteworthy finding, though it is unclear whether a similar effect is observed at institutions where a more significant proportion of students intercalate, and the effect of differing course structures also warrants investigation. In addition, there have been no investigations on the effect of intercalating on the foundation programme application process.

We therefore set out to address these areas by exploring the effect of doing an intercalated BSc on subsequent academic performance and Foundation School outcome, at an institution where the majority of students do intercalate, with the additional options of being able to intercalate elsewhere and/or choosing to do so in the later clinical years.

## Methods

### Subjects

The study subjects were medical students at King's College London that graduated in the years 2007 to 2009. This included students on the standard 5-year programme, graduates on the accelerated 4-year course (GPEP), and students on a 6-year extended programme (EMDP). Students are offered the choice to do an intercalated BSc between MBBS years 2 and 3, years 3 and 4, or years 4 and 5. The majority of students at this institution do intercalate, primarily between their second and third year of medical studies.

### Assessment

Final examination results for each year of medical education were collated in Microsoft Excel. Information on whether students intercalated, when and where they chose to do so (internally at King's College London or at an external institution), their choice of subject and their degree classification were also collected from student records. In addition, details of the course type (as detailed above), graduation year and those that were graduates were also recorded.

Data on overall Foundation School scores from two successive cohorts were also analysed. This score is comprised of the academic quartile score and the application form score. The academic quartile is determined by the performance on exams, while the latter is achieved through answering white space questions. The academic quartile score and the application form score that students achieved were also recorded. Data from the Foundation School preference students achieved were also recorded and analysed.

### Statistical Analysis

Stata/MP 11 for Windows was used for data storage and analysis. Univariate analysis was conducted to explore the relationship between intercalating and other factors, without adjusting for possible confounders. Proportions were compared using chi-squared or Fisher's exact tests as appropriate, and mean exam results in each year of study were compared using one-way ANOVA's. Multivariable linear regression analysis was used to model the relationship between exam performance and intercalation. Year 5 result was the primary outcome and the model was adjusted for baseline (Year 1) exam results, graduate status, year of graduation, and course type. Further linear regression models were fit, first to only those who intercalated to examine the effect of time of intercalation on future performance,, and secondly to only those intercalating between years 2 and 3 to examine the effect of intercalating on year 3, 4 then 5 results while adjusting for all previous years exam results.

Using data from two cohorts, linear regression analysis was subsequently used to investigate the impact intercalating had on overall Foundation School score and on application score. This was again adjusted for baseline (Year 1) exam results, graduate status, year of graduation, and course type. An ordered logistic regression model was used to examine the association between intercalating and the academic quartile, and then intercalating and the choice of Foundation School that students achieved. The same variables outlined above were adjusted for with these models. Further models were also fit including all examination results obtained prior to the calculation of the outcome.

Finally, the associations between intercalated degree classification and each of the above outcomes were investigated by including degree classification in appropriate models for students who intercalated internally (data not available for external degrees).

Ethical approval was not required as this was a retrospective analysis of an anonymised database.

## Results

The study included 1158 students: 346 who graduated in 2007, 402 from 2008, and 410 students that graduated in 2009. Overall, the majority of these students were on the standard 5-year programme (1085; 93.7%), there were 49 graduates who had completed a degree prior to their medical training (9.2%), and 24 students (2.1%) on the extended 6-year medical programme. The majority of students intercalated (625; 54.0%), with 61 of these students (9.8%) choosing to do so at an external institution. In the years being investigated, 200 (57.8%), 209 (52.0%) and 216 (52.7%) students intercalated from the 2007, 2008 and 2009 graduating cohorts respectively.

Table 1 provides comparison data for non-intercalating students (n = 533), intercalated students who remained at the institution (n = 564), and those that intercalated elsewhere (n = 61).

**Table 1:** Univariate analysis of students that: did not intercalate, intercalated internally, and those that intercalated elsewhere.

	Total (n = 1158)	Did not Intercalate (n = 533)	Intercalated: Internal (n = 564)	Intercalated: External (n = 61)	P-value
Course, n(%)					
Medicine MBBS	1085	465 (42.9)	560 (51.6)	60 (5.5)	P < 0.0001
Extended MBBS	24	19 (79.2)	4 (16.7)	1 (4.2)	
Graduate MBBS	49	49 (100)	0 (0.0)	0 (0.0)	
Year, n(%)					
2006/7	346	146 (42.2)	180 (52.0)	20 (5.8)	P = 0.539
2007/8	402	193 (48.0)	190 (47.3)	19 (4.7)	
2008/9	410	194 (47.3)	194 (47.3)	22 (5.4)	
Graduate, n(%)					
No	847	227 (26.8)	559 (66.0)	61 (7.2)	P < 0.0001
Yes	311	306 (98.4)	5 (1.6)	0 (0.0)	
Year of Intercalation, n(%)					
Between Yr 2&3	496	n/a	460 (92.7)	36 (7.3)	P < 0.0001
Between Yr 3&4	73	n/a	58 (79.5)	15 (20.5)	
Between Yr 4&5	47	n/a	38 (80.9)	9 (19.2)	
Exam Results, mean(sd)					
Year 1	67.0 (7.3)	68.3 (7.8)	66.3 (6.7)	64.3 (6.7)	P < 0.00001
Year 2	66.1 (6.4)	66.6 (6.9)	65.9 (6.2)	64.3 (5.2)	P = 0.0175
Year 3	73.0 (6.6)	73.2 (6.6)	73.0 (6.6)	72.0 (6.7)	P = 0.3816
Year 4	68.4 (5.8)	68.6 (6.0)	68.4 (5.6)	66.4 (6.0)	P = 0.0225
Year 5	69.2 (5.8)	69.3 (5.8)	69.2 (5.7)	67.9 (6.2)	P = 0.1684
Foundation Score	71.1 (9.4)	70.4 (10.2)	71.8 (8.6)	70.0 (9.4)	P = 0.1399
Application Score	34.1 (7.3)	33.1 (7.8)	35.0 (6.7)	33.7 (8.0)	P = 0.0024
Quartile, n(%)					P = 0.1660
Quartile 1 (top)	186	80 (23.5)	95 (26.3)	11 (31.4)	
Quartile 2	188	77 (22.6)	99 (27.4)	12 (34.3)	
Quartile 3	194	92 (27.0)	95 (26.2)	7 (20.0)	
Quartile 4	170	92 (27.0)	73 (20.2)	5 (14.3)	

There was a significant difference between the proportions of students who decided to intercalate on the three medical courses (P < 0.0001). Intercalated students were most commonly from the 5-year programme, with 57.1% of these students intercalating. Of the students that did not intercalate a significant proportion were graduates (P < 0.0001), with only 5 students choosing to do so, all of whom belonged to the standard 5-year course. There was no significant variation between the proportion of students that intercalated from the successive cohorts (P = 0.539).

Multivariate analyses examining the effect of intercalating on Year 5 results are displayed in Table 2. It shows that, following adjustment for course type, year of graduation, graduate status and baseline (Year 1) performance, that doing an intercalated BSc led to a statistically significant improvement in subsequent exam performance, as measured by the Year 5 result. Internally intercalating students had a mean year 5 result that was on average 1.27 points (95% CI 0.52 to 2.02) greater than those who did not intercalate while externally intercalating students scored 0.79 points (95% CI -0.58 to 2.16) higher.

**Table 2:** Multivariate linear regression analysis of student exam performance.

Year 5 result as outcome	Coefficient	95% Confidence Interval	P-value
Course			P < 0.00001
Medicine MBBS	Ref	Ref	
Extended MBBS	-4.25	(-6.30 to -2.21)	
Graduate MBBS	1.97	(0.42 to 3.52)	
Year			P < 0.00001
2006/7	Ref	Ref	
2007/8	-1.80	(-2.54 to -1.06)	
2008/9	-0.81	(-1.54 to -0.08)	
Year 1 result	0.39	(0.35 to 0.43)	P < 0.00001
Graduate			P = 0.6334
No	Ref	Ref	
Yes	0.23	(-0.73 to 1.19)	
Intercalation			P = 0.0041
Did not Intercalate	Ref	Ref	
Intercalated: Internal	1.27	(0.52 to 2.02)	
Intercalated: External	0.79	(-0.58 to 2.16)	

Year 5 result was used as the outcome to examine the effect of intercalation. The model was adjusted for baseline Year 1 results, graduate status, year of graduation, and course type.

It was also found that students on the extended medical course (EMDP) attained Year 5 results which were on average over 4 points lower than students on the regular 5-year programme (95% CI -6.30 to -2.21). In addition, students on the graduate course (GPEP) were found to score on average 1.97 points higher than the students on the standard course (95% CI 0.42 to 3.52).

Time of intercalation was not found to be associated with year 5 performance (p = 0.3096) when a model was fit to only intercalating students. Further analyses were carried out including only those who intercalated between years 2 and 3, or not at all. After adjusting for year 1 and year 2 results and other covariates, intercalating was significantly associated with improved year 3 results (P < 0.0001). Further, there was a significant improvement in year 4 results when year 3 was included in the model (P < 0.0001) and in year 5 results when year 3 and 4 were both included (data not shown).

To explore whether this observation that doing an intercalated BSc led to statistically significant higher exam results, had further benefits, the association with the foundation application process was investigated. Examining two cohorts for which data were available, Table 3 ('Overall Foundation Programme' column) shows that doing an intercalated BSc led to a higher overall score on the foundation programme application (P < 0.00001), following adjustment for baseline (Year 1) results, graduate status, year of graduation, and course type. Students who intercalated internally had a score on average 6.07 points higher (95% CI 4.52 to 7.62) than those that did not intercalate. Externally intercalating students also had improved scores, which were on average 4.94 points higher (95% CI 2.07 to 7.82).

**Table 3:** Multivariate linear regression analysis of student performance on overall foundation programme score and application score.

	Overall Foundation Programme Score			Application Score			Quartile Score		
	Coefficient	95% CI	P-value	Coefficient	95% CI	P-value	Odds Ratio	95% CI	P-value
Course			P = 0.0010			P = 0.0276			P = 0.0002
Medicine MBBS	Ref	Ref		Ref	Ref		Ref	Ref	
Extended MBBS	-2.38	(-5.96 to 1.19)		-0.77	(-3.59 to 2.05)		0.29	(0.11 to 0.71)	
Graduate MBBS	4.80	(2.09 to 7.51)		2.86	(0.72 to 4.99)		2.98	(1.47 to 6.06)	
Year			P < 0.00001			P < 0.00001			P = 0.9619
2007/8	Ref	Ref		Ref	Ref		Ref	Ref	
2008/9	6.47	(5.29 to 7.64)		6.62	(5.69 to 7.54)		0.99	(0.75 to 1.32)	
Year 1 result	0.44	(0.35 to 0.53)	P < 0.00001	0.16	(0.09 to 0.22)	P < 0.00001	1.19	(1.16 to 1.22)	P < 0.00001
Graduate			P = 0.0001			P = 0.0001			P = 0.3746
No	Ref	Ref		Ref	Ref		Ref	Ref	
Yes	3.95	(1.95 to 5.96)		3.15	(1.57 to 4.73)		1.24	(0.77 to 2.01)	
Intercalation			P < 0.00001			P < 0.00001			P = 0.0003
Did not Intercalate	Ref	Ref		Ref	Ref		Ref	Ref	
Intercalated: Internal	6.07	(4.52 to 7.62)		4.59	(3.36 to 5.81)		2.18	(1.48 to 3.19)	
Intercalated: External	4.94	(2.07 to 7.82)		3.38	(1.11 to 5.64)		2.06	(1.02 to 4.16)	

Overall score (comprised of academic quartile score and application form score) was used as the outcome to examine the effect of intercalation. This was followed by application score as the outcome. Ordered logistic regression analysis of student performance was used for quartile score. Quartile score (from exam results and subsequent position in cohort) was used as the outcome for this analysis. The model was adjusted for baseline Year 1 results, graduate status, year of graduation, and course type.

To determine whether this difference was a result of differences in the academic quartile or application form scores, or both, each was used as an outcome. Table 3 ('Application Score' column) shows the effect on the application form score in the various groups, following adjustment for the same variables outlined above. Internal students were found to have application scores which were on average 4.59 points higher (95% CI 3.36 to 5.81) than students that did not intercalate and students that intercalated externally also had higher scores, which were on average 3.38 points higher (95% CI 1.11 to 5.64) than their non-intercalating colleagues (P < 0.00001). The associations between foundation and application scores and intercalation status remained statistically significant when adjusted for all exam results from Year 1 to Year 5.

Using an ordered logistic regression model, Table 3 ('Quartile Score' column) shows that, following adjustment for the above variables, intercalated students also had a statistically significant higher academic ranking compared to those that did not intercalate (P = 0.0003). It was found that for internally intercalating students, the odds of being in a higher group were 2.17 times higher (95% CI 1.48 to 3.19) than if a student did not intercalate. Students that intercalated externally had odds which were 2.06 higher (95% CI 1.02 to 4.16).

Doing an intercalated BSc therefore led to a statistically significant increase in the overall score, which was observed in both the quartile ranking and score on the white-space questions on the application form. To determine whether these observations actually led to greater success with securing foundation jobs, the association with the Foundation School preference students achieved was explored.

Table 4 shows the odds of securing the first choice Foundation School in those who internally and externally intercalated compared to those that did not intercalate. Students that intercalated had an increased odds of getting into their first choice Foundation School (P = 0.0220) with the odds for those internally intercalating 2.18 times higher (95% CI 1.20 to 3.98) than those that did not intercalate. In students that intercalated externally, the odds ratio was 0.96 (95% CI 0.37 to 2.53), and suggests that these students had similar odds to those that did not intercalate.

**Table 4:** Ordered logistic regression analysis of student performance on Foundation School preference awarded.

Foundation School Preference as outcome	Odds Ratio	95% Confidence Interval	P-value
Course			P = 0.8224
Medicine MBBS	Ref	Ref	
Extended MBBS	1.00	(0.27 to 3.70)	
Graduate MBBS	1.61	(0.33 to 7.80)	
Year			P = 0.0357
2007/8	Ref	Ref	
2008/9	0.58	(0.35 to 0.97)	
Year 1 result	1.07	(1.03 to 1.11)	P = 0.0003
Graduate			P = 0.3933
No	Ref	Ref	
Yes	1.44	(0.61 to 3.39)	
Intercalation			P = 0.0220
Did not Intercalate	Ref	Ref	
Intercalated: Internal	2.18	(1.20 to 3.98)	
Intercalated: External	0.97	(0.37 to 2.53)	

Choice of Foundation School awarded was used as the outcome to examine the effect of intercalation. The model was adjusted for baseline Year 1 results, graduate status, year of graduation, and course type.

After adjustment for previous exam results and sample characteristics, there was no association between classification of intercalated degrees and Year 5 exam results (p = 0.7049), overall foundation score (p = 0.0689), application form score (p = 0.3149) or academic quartile ranking (p = 0.488) (data not shown).

## Discussion

This study aimed to determine the benefits of intercalating on academic performance and career progression. Higher exam results at medical school would lead to an improved academic ranking and result in a greater overall score on the foundation programme application. This is beneficial as it would give students greater leverage with securing starting posts in their desired specialities. Since the foundation posts form an important step in influencing an individual's choice of final specialty, any advantage that can be conferred during medical school would be significant.

This study found that intercalated students had higher subsequent exam results, compared to those that did not intercalate. Intercalated students had improved results from the year after intercalating until the end of their medical school career, even after adjusting for results from previous years. Further, this translated to higher overall scores on the foundation application. When determining whether this increase in final score was due to the academic ranking or points from the white space questions, it was found that there was actually a statistically significant increase in both of these sections of the application. Therefore, intercalated students performed significantly better in multiple areas of the form, assessing a diverse range of proficiencies. These improvements were more pronounced in those that remained at the institution to intercalate. In particular, the students that intercalated internally had greater success with securing their first choice Foundation School, compared to those that did not intercalate. Students that intercalated externally did not have improved odds of achieving their choice of Foundation School compared to those that did not intercalate.

The improvement in subsequent exam results in students that intercalated may be due to the acquisition of new and more suitable learning styles [8]. The analytical and organisational skills that students learn during their intercalated year may be of benefit with the more demanding exams in the final years of the medical course. The intercalated year may also facilitate more self-directed learning, which is in contrast to the largely didactic teaching prevalent on medical programmes nationally. This allows students to think more independently and encourages lateral thought. In addition, examinations in the intercalated year are essay-based and under timed conditions, requiring students to develop the ability to express themselves succinctly and precisely. These skills may be of benefit with the white space questions on the foundation application and may explain the scores observed in intercalated students. Students who intercalated at the institution also had greater success with securing their first choice Foundation School. Since many students intercalate for the purposes of career progression [2], this indicates that doing so is indeed beneficial. Further work would help to elucidate whether students secure their chosen specialities in the subsequent round, following the allocation of Foundation Schools.

In general, students who intercalated externally did not show the same degree of improvement with all the outcomes that were measured. These students may have greater difficulty readjusting to the medical programme, following a year away. The year that these students spend away exposes them to a new environment, with access to different learning resources from the ones that they may be accustomed to. These additional adjustments may hinder their settlement, preventing them from optimising their learning during their intercalated degree. These students may therefore have a greater challenge advancing the skills that they would be expected to develop during this year compared to students who remain at the institution, who avoid such difficulties. These observations may also be due to varied intercalated degree structures and subject choices at other institutions. Only 25.3% of students who intercalated internally chose to study non-traditional BSc subjects. As degree subject was not available for those intercalating externally it was not possible to make comparisons between the range of degrees studied internally and externally. However, if those externally intercalating favoured non-traditional subjects, with less focus on basic medical science, this may partially explain the lower performance in future examinations compared to those who intercalated internally.

Surprisingly, external intercalating students did not show the same degree of success with their Foundation School allocations, not achieving their first choice post. Nonetheless, these students also had improvements in overall foundation scores, and so this finding may be due to the competition for places at the specific Foundation Schools where applications were made by these students. In addition, given the relatively fewer students that intercalated externally, a study involving more cohorts would help to fully explore this relationship.

Interestingly, some differences were noted between the conventional, EMDP and GPEP students, with GPEP students achieving higher average scores in all medical school exams and in the foundation school application process. The stronger academic performance of GPEP students may be attributed to greater maturity in this group, rather than the benefits of having a prior degree [9]. EMDP students had a weaker performance compared to both graduates and students on the conventional course. This may be related to the reduced entry requirements for the extended medical programme and other socioeconomic differences.

Intercalated students were observed to have slightly lower year 1 and year 2 results. In a recent study, it was found that students who intercalated after their second year exams had higher results before choosing to intercalate [10]. However, in this study differences exist among those who do and do not intercalate, which led to higher raw scores in the intercalating group; graduates, the majority of which did not intercalate, had higher scores than non-graduates, which skews the baseline exam results.

Using three large successive cohorts this study showed favourable outcomes from doing an intercalated BSc, at an institution where the majority of students intercalate. While the results of externally intercalating students showed trends towards improvement, the fact that some of these were not significant may be attributed to the relatively small number of students that belonged to this group. A larger sample would be required to provide sufficient power to fully explore the differences between internally and externally intercalating students. Other factors which were not considered in this study may also have been significant, such as the gender and age of students, as well as degree classification. End of year results comprise of a number of assessments which test both practical and theoretical skills, and a breakdown of the individual assessments within each medical year may also have been of interest.

While intercalating significantly improved subsequent exam performance, further work would also help to determine whether these benefits correlate with long-term career prospects, specifically with competitive speciality training and careers in academia. Indeed, studies have shown that medically qualified professors and readers were more likely to have an intercalated degree and those with such a degree had greater success obtaining research grants [1]. Additional work may also objectively determine the effect of intercalating on work in the clinical setting, assessing the ability to critically appraise new research and practise evidence-based medicine. Further work will also help to elucidate whether the improvement in academic performance and Foundation School preference will translate to the proposed Situational Judgement Testing (SJT) that is being piloted. With national implementation of the SJT approach to selection, this investigation can be repeated once data is available.

## Conclusion

Intercalating students perform better in subsequent exams. This is associated with improved overall foundation scores, due to an increase in both academic quartile points, and application score. This translates into greater success with Foundation School posts in students, particularly apparent in the internally intercalating cohort. These differences may subsequently be invaluable in securing posts in students' desired specialities. These factors should be considered by prospective intercalating students.

## Competing interests

The authors declare that they have no competing interests.

## Authors' contributions

NM was involved in the conception and design of the study, interpretation of data, drafting of manuscript and approved final version. SC was involved in acquisition of data and its analysis, reviewing manuscript and approving the final version. HS was involved in acquisition of data, guidance on interpretation of data, reviewing manuscript and approving the final version. SH was involved in the conception and design of the study, reviewing manuscript and approval of final version.

## Pre-publication history

The pre-publication history for this paper can be accessed here:

http://www.biomedcentral.com/1472-6920/11/76/prepub
